# Diagnostic features of tuberculous meningitis: a cross-sectional study

**DOI:** 10.1186/1756-0500-5-49

**Published:** 2012-01-20

**Authors:** Paul Matthew Pasco

**Affiliations:** 1 Dept of Neurosciences, UP-PGH Medical Center, Taft Ave, Manila, Philippines

## Abstract

**Background:**

Tuberculous meningitis (TBM) is a common central nervous system infection in the Philippines; however it is difficult to diagnose as findings are non-specific. Hence we decided to determine if, among patients with chronic meningitis syndrome, the following are associated with the diagnosis: new-onset seizures; focal neurologic deficit; pulmonary tuberculosis (PTB) on chest X-ray; cerebrospinal fluid (CSF) pleocytosis with lymphocytic predominance; decreased CSF glucose; increased CSF protein.

**Methods:**

Adult patients with suspected TBM were enrolled after informed consent was obtained. Baseline physical examination and diagnostic tests including CT scan of the head with contrast and CSF analysis for acid fast bacilli (AFB) smear, TB culture and cryptococcal antigen detection were done and results collected. Definite TBM was defined as positive AFB smear or positive TB culture or positive basal meningeal enhancement on CT contrast study. Logistic regression was done to determine which were associated with a diagnosis of TBM.

**Results:**

91 patients were included. Using the gold standard criteria mentioned above, 44 had definite TBM; but if subsequent clinical course and response to anti-Koch's therapy are considered, 68 had a final diagnosis of TBM. After logistic regression was performed, only abnormal CSF (the combination of CSF pleocytosis with lymphocytic predominance, decreased CSF glucose, and increased CSF protein) was associated with the diagnosis of TBM.

**Conclusion:**

In patients with chronic meningitis syndrome, only abnormal CSF was associated with the diagnosis of TBM.

## Background

Tuberculous meningitis (TBM) is the most dreaded manifestation of tuberculosis, and is a common infection of the central nervous system (CNS) especially in developing countries like the Philippines where tuberculosis is highly endemic. The incidence follows that of pulmonary tuberculosis, and is associated with high mortality and morbidity [1]. In a large-scale epidemiological study of extrapulmonary tuberculosis in the United States, CNS involvement was noted in 5-10% of extrapulmonary tuberculosis cases, with more recent CDC data in 2005 indicating that 6.3% of extrapulmonary cases (1·3% of total tuberculosis cases) involve the CNS [2].

Tuberculosis in the Philippines ranks sixth among the top causes of morbidity and mortality, with rates of 134·1/100,000 and 31·2/100,000 respectively in 2005. As for TB of the CNS, based on partial prevalence data from eight Metro Manila hospitals, TBM is the most common primary CNS infection in adults in the country, accounting for 28·9% of all cases of primary CNS infections [3]. In the Philippine General Hospital the case fatality rate for CNS infections is 20%, and prognosis depends on how soon a diagnosis is made and appropriate therapy is started.

The diagnosis of TBM remains to be difficult. As with other forms of TB the gold standard is isolation of the organism through culture or detection of its presence by acid-fast staining. However the yield from acid-fast staining and culture remains to be very low, probably because of the low concentration of bacilli in the cerebrospinal fluid (CSF). The author's own series of 63 cases had a positive culture yield in only 3 of these [4]. Molecular methods, such as polymerase chain reaction (PCR)-based diagnostic techniques, have begun to be applied in the local setting, but are relatively expensive, and the yield greatly depends on stringent quality control. Ultimately clinicians still rely on indirect evidence of TBM, such as the changes in the cerebrospinal fluid, as well as the absence of another likelier diagnosis. Hence the search continues for a reliable, accurate, yet easily performed and inexpensive diagnostic test.

Due to the unsatisfactory nature of current diagnostic methods, some have formulated clinical decision rules to determine the likelihood of a diagnosis of TBM using only clinical and simple laboratory findings. Thwaites et al. [1] compared the clinical and laboratory features of 251 Vietnamese adults with either tuberculous or bacterial meningitis. Five features were independently associated with a diagnosis of tuberculous meningitis: age, length of history, white blood cell count, total cerebrospinal fluid (CSF) white cell count, and CSF neutrophil proportion. However this study included patients with bacterial meningitis, which is often not an important differential diagnosis in patients with TBM. More recently, Moghtaderi et al. [5] retrospectively compared clinical and laboratory features in 68 Iranian patients with TBM and 123 cases of acute bacterial meningitis. Disease duration of ≤ 5 days, age over 30 years, CSF leukocytosis and lymphocytosis were independent predictive factors for a diagnosis of TBM.

The common limitation of these researches is that not enough patients with a chronic meningitis syndrome were included in their patient populations. Patients with TBM were usually compared to patients with acute bacterial meningitis, when clinically this problem is not often encountered. It is frequently more crucial to differentiate between the different causes of a chronic meningitis syndrome, such as cryptococcal meningitis. Tests to rule out this important differential diagnosis were apparently not carried out in the above studies mentioned. These shortcomings are addressed in the present study.

While the ideal diagnostic test is still to be developed, clinicians need to know which features are associated with the diagnosis of TBM, so that appropriate therapy can be started early, and the toxicities of unnecessary treatment can be avoided, hence improving the likelihood of a good outcome for patients with this devastating illness. The results of this study will hopefully contribute to determining which clinical and simple laboratory features are associated with the diagnosis in the local setting.

**Figure 1:**
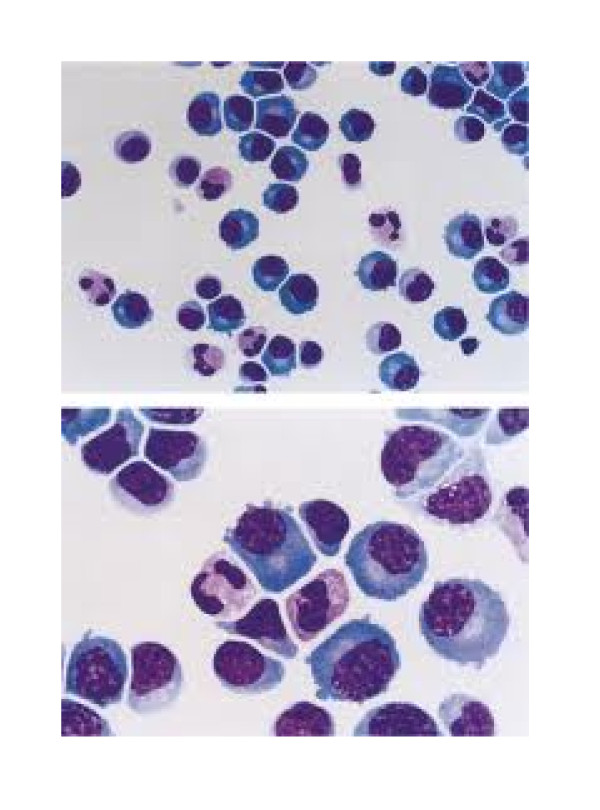
**CSF pleocytosis with lymphocytic predominance in abnormal CSF smears**.

This study aimed to determine if the following clinical and laboratory features are associated with a diagnosis of definite TBM in patients with chronic meningitis syndrome: new-onset seizures; focal neurologic deficit; (+) PTB on chest X-ray; CSF lymphocytic pleocytosis (predominance of lymphocytes rather than neutrophils, which is more characteristic of a chronic infection such as TBM, as in Figure 1); decreased CSF glucose; increased CSF protein.

## Methods

**Figure 2:**
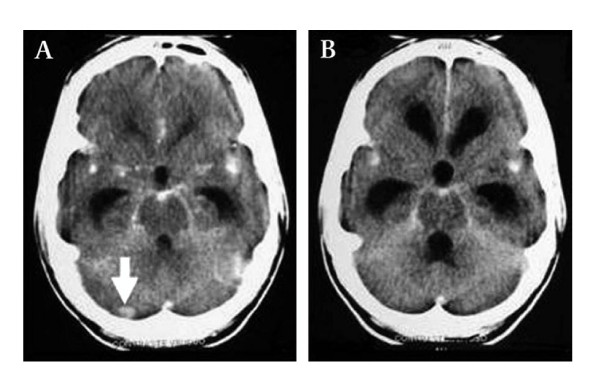
**CT scan of head showing basal meningeal enhancement on contrast study**. Arrow points to a tuberculoma.

This is a prospective cross-sectional validity study carried out at the Philippine General Hospital. The operational definitions adopted for this study are as follows:

***a*. Definite Tuberculous meningitis (TBM): **Any patient whose cerebrospinal fluid (CSF) is positive for AFB smear *or *positive for TB culture *or *with basal meningeal enhancement on CT head contrast study (Figure 2).

**b. Chronic meningitis syndrome: **any patient with 7 days or more history of any 2 of the following: headache (as subjectively reported by the patient or family member); fever (any subjective report of increased body temperature); sensorial change.

**c. Sensorial change: **change in the level of sensorium from normal awake to decrease (drowsiness/stupor/coma) or increase (delirium/restlessness).

**d. New-onset seizure: **development of any type of seizure since the start of the patient's present illness.

**e. Focal neurologic deficit: **development of any of the following since the start of the patient's present illness: dysphasia, dyspraxia, agnosia, dyscalculia, left-right disorientation, cranial nerve deficit such as anosmia, dysarthria, weakness of one or more limbs, sensory deficit in one or more limbs, dysmetria, ataxia.

**f. Pulmonary TB: **chest X-ray interpreted as pulmonary TB by a radiologist.

**g. Basal meningeal enhancement on CT head contrast study: **marked contrast enhancement outlining the basal cisterns on head CT, as interpreted by a radiologist.

**h. CSF pleocytosis with lymphocytic predominance: **presence of more than 50 white cells/mm^3 ^on microscopic examination of the CSF, with more lymphocytes than polymorphonuclear cells (Figure 1).

**i. Decreased CSF glucose: **value of CSF glucose 50% or less than simultaneous serum glucose determination.

**j. Increased CSF protein: **value of CSF protein more than the upper limit of normal of the performing laboratory's reference values, i.e. > 0.45 mg/dl.

**k. Abnormal CSF: **presence of all 3 of the above CSF findings (CSF pleocytosis with lymphocytic predominance + decreased CSF glucose + increased CSF protein.

Prior to carrying out this study, ethical approval was obtained from the Research Implementation and Development Office of the University of the Philippines College of Medicine.

Any adult patient with any two of headache, fever, or sensorial change, of at least 7 days duration, and without any other apparent etiology of the illness, was considered to have chronic meningitis syndrome and included in the study after appropriate informed consent was obtained. The following baseline diagnostic tests were performed on all enrolled patients: cranial CT scan with and without contrast; chest X-ray; and lumbar puncture for CSF analysis. Aliquots of the CSF obtained were sent for the following examinations: 2-3 cc for AFB smear and TB culture (Bactec method), 2-3 cc for routine bacteriologic Gram stain & culture, and 1-2 cc for India ink stain & cryptococcal latex agglutination test (CALAS). Patients were treated according to the results of the above examinations, and the best clinical judgment of the attending physician. Data on the clinical outcome and final etiologic diagnosis upon discharge were collected.

All data were entered using Excel software in an Excel file. Descriptive statistics for demographic variables were calculated; crude odds ratios for the presence of each of the above clinical features were then obtained, and logistic regression was performed.

## Results

A total of 91 adult patients with chronic meningitis syndrome were enrolled in the study from July 2005 to June 2008, with an age range of 19-76 years old (mean 35·3 years old, s. d. 14·0) and male: female ratio of 1·53:1. Of these 91 patients, 44 were diagnosed to have definite TBM according to the composite reference standard (positive for AFB smear *or *positive for TB culture *or *with basal meningeal enhancement on CT head examination, as in Figure 2).

If subsequent clinical course, follow-up data, results of other laboratory examinations, and response to anti-Koch's therapy are taken into account, 68 had a final diagnosis of TBM upon discharge based on the attending physician's clinical assessment, while 22 had other alternative diagnoses (Table 1).

**Table 1:** Final diagnosis in 91 study patients

Final Diagnosis	Frequency (%)
TB meningitis	68 (74·7)

Bacterial meningitis	6 (6·6)

Viral encephalitis	6 (6·6)

Septic encephalopathy	3 (3·3)

Cryptococcal meningitis	2 (2·2)

Metabolic Encephalopathy	2 (2·2)

Hypoxia	1 (1·1)

Normal pressure hydrocephalus	1 (1·1)

Subarachnoid hemorrhage	1 (1·1)

No final diagnosis	1 (1·1)

**Grand Total**	**91 (100%)**

To find out which clinical and laboratory features were associated with a diagnosis of TBM, we performed univariate analysis of the data using simple logistic regression (Table 2). Taken individually, lymphocytic pleocytosis, decreased glucose, or increased protein, would seem to be significant predictive factors of a diagnosis of TBM.

**Table 2:** Univariate analysis of potential factors associated with a diagnosis of definite TBM; definite TBM = 44; not definite TBM = 47

Factor	(+) in definite TBM (n = 44)	(-) in definite TBM (n = 44)	Crude odds ratio	**95% C. I**.	P value
**New-onset seizure**	14	13	1·04	0·38-2·86	0·93

**New-onset neurologic deficit**	24	21	0·45	1·19-2·93	0·76

**Presence of PTB**	15	10	1·72	0·60-4·98	0·26

**Lymphocytic pleocytosis**	32	18	3·56	1·32-9·74	0·005

**Decreased glucose**	33	21	3·00	1·10-8·30	0·017

**Increased protein**	40	30	4·89	1·12-24·43	0·015

After logistic regression, however, these 3 factors were no longer significant. But since these 3 CSF features were initially significant on univariate analysis, and in clinical practice they are usually considered together, we decided to combine them to determine if this new variable would be significant after multiple regressions. Accordingly, the new variable was named "abnormal CSF," and a patient was classified to have abnormal CSF if the patient had all 3 CSF features (lymphocytic pleocytosis, decreased glucose, increased protein).

Univariate analysis was then repeated for all the four features (abnormal CSF, new-onset seizure, new-onset deficit, and (+) PTB on CXR.) The results are shown in Table 3. The new variable, abnormal CSF is now the only factor which turns out to be significant after univariate analysis.

**Table 3:** Univariate analysis of potential factors associated with definite TBM; definite TBM = 44; not definite TBM = 47

Factor	(+) in definite TBM (n = 44)	(-) in definite TBM (n = 44)	Crude odds ratio	**95% C. I**.	P value
New-onset seizure	14	13	1·04	0·38-2·86	0·93

New-onset neurologic deficit	24	21	1·14	0·45-2·93	0·76

Presence of PTB	15	10	1·72	0·60-4·98	0·26

Abnormal CSF	26	13	3·78	1·44-10·04	0·002

After logistic regression analysis, abnormal CSF remained significant (Table 4). For this overall logistic regression model, the likelihood ratio statistic is 8·80, with a *p*-value of 0·06. Given this result of near significance at an alpha level of 0·05, it would be helpful to proceed in assessing the effect of the predictor variables when taken together in the diagnosis of TBM.

**Table 4:** Overall Logistic Regression of possible predictive factors of TBM

Factor	Odds ratio	**95% C. I**.	P value
New-onset seizure	1·49	0·54-4·07	0·44

New-onset neurologic deficit	1·17	0·47-2·90	0·72

Presence of PTB	2·06	0·74-5·72	0·17

Abnormal CSF	3·27	1·30-8·22	0·01

In order to further assess the significance of abnormal CSF as well as the other factors, the likelihood ratio test was performed to identify factors associated with the diagnosis of TBM. At the end of the LR test only abnormal CSF was found to be a significant associated factor for the diagnosis of TBM. Since only one factor was significant, an ROC curve was no longer constructed; instead the validity parameters of abnormal CSF as a diagnostic test for definite TBM were calculated. The results, shown in Table 5, reveal only a moderate sensitivity and specificity, and likelihood ratios:

**Table 5:** Validity parameters of abnormal CSF for the diagnosis of TBM

Parameter	Value	**95% C. I**.
**Sensitivity**	0·57	0·42-0·71

**Specificity**	0·69	0·55-0·81

**Likelihood ratio (+)**	1·89	1·12-3·20

**Likelihood ratio (-)**	0·61	0·41-0·91

## Discussion

In this study, clinical and widely available laboratory features were collected from 91 patients with a chronic meningitis syndrome to find out which of these were associated with a diagnosis of TBM. Based on these test results (positive for TB culture or AFB smear or basal meningeal enhancement on contrast CT scan of the head), only 44 would fulfil the criteria of definite TBM. If only growth on TB culture or positive AFB smear were used as the gold standard, then this number would be even lower (4/91). The final discharge diagnosis was TBM in 68 of the 91 cases, whereas there were other alternative diagnoses in the other cases. Other CNS infections were among the next most common final diagnoses: bacterial and viral meningitis (6·6% each) and cryptococcal meningitis (2·2%). Non-infectious causes (6/91) were also among the differential diagnoses, such as metabolic encephalopathy, hypoxia, and subarachnoid hemorrhage, but these are usually easily ruled out on the basis of history, PE, and simple laboratory examinations.

Due to the low yield of conventional TB culture or AFB smear in our setting, we chose to include basal meningeal enhancement on CT contrast study of the head as part of the gold standard criteria for TBM. Several authors have determined the validity of this approach: Kumar (1996) compared the CT findings of 94 children with TBM with those of 52 children with pyogenic meningitis, and concluded that basal meningeal enhancement, tuberculoma, or both, were 89% sensitive and 100% specific for the diagnosis of TBM. Przybojewski [5] showed that the criteria most clinically useful were the Y-sign, linear enhancement, contrast filling the cisterns, and asymmetry, due to their high likelihood ratios.

Due to the shortcomings of the laboratory examinations used as the gold or reference standard in this study, an alternate reference standard could have been used, such as a consensus panel diagnosis, wherein at least two experts would examine each chart and arrive at a consensus as to whether each patient did indeed have TBM. Important data such as the response to anti-Koch's medications could have been taken into account for the panel to arrive at a diagnostic decision with a confident level of certainty. However the panel would have to be blinded to the results of the tests being studied as possible predictive factors, lest incorporation bias come about and artificially inflate the odds ratios.

It is unfortunate that only abnormal CSF was found to be associated with the diagnosis of TBM, in contrast to previous studies. Thwaites, Kumar, Sunbul, Youssef, and Moghtaderi [1,6-9], cited in the review of literature. This may be because of differences in the study population. In the earlier studies, the population was of patients presenting as a meningitis syndrome, whether acute or chronic; cases of TBM were compared with other bacterial meningitides, but arguably this would not be useful in an actual clinical setting because it is not often that clinicians have to discriminate between TBM and acute bacterial meningitis. More often the dilemma is to distinguish between TBM and other chronic meningitides such as cryptococcal meningitis. Also, the population in the present study would be more relevant for local clinicians, as the clinical presentation of TBM in the country may be different from places such as Vietnam and India, places with high HIV prevalence, where the previous studies were carried out. Coinfection with HIV increases the risk of TBM and modifies the clinical presentation: usually patients would present with acellular CSF and high AFB loads [6].

This study shows that diagnostic rules and prediction models which were developed in one setting may not necessarily be relevant in another setting, as the factors which were found relevant in previous studies turned out not to be significant in the local setting, most probably due to differences in the study population.

This study also shows that an abnormal CSF profile (lymphocytic pleocytosis + decreased glucose + increased protein) has a moderate sensitivity and specificity, in fact comparable with more advanced molecular methods such as PCR, and a definite improvement over the current gold standard of AFB smear or TB culture. Hence in the setting of a chronic meningitis syndrome, clinicians confronted with this finding may be reasonably confident of the diagnosis of TBM, and use the associated likelihood ratios to arrive at a decision as to whether to order more tests or proceed with anti-Koch's treatment. Also, clinicians confronted with these findings should first rule out those conditions which have entirely different therapies, such as cryptococcal meningitis and partially-treated bacterial meningitis, by doing the appropriate laboratory exams. In those patients where it is not immediately evident what the etiologic organism is, then the best course would be to treat for TBM and start anti-Koch's medications by default, as this is the most prevalent etiology of a chronic meningitis in the local setting, and then to also institute additional treatment for those other conditions which the clinician is ruling in. He may then modify his subsequent management based on the response to therapy, and a repeat of the pertinent laboratory exams where appropriate.

There are some limitations to this study:

1) The desired sample size was not achieved within the time frame of this study; conceivably some factors would turn out to be positively predictive if a larger population were studied;

2) There is subjectivity involved in assessing for the presence of basal meningeal enhancement on CT head contrast study; also inter-rater and intra-rater variability was not assessed. This would have affected the performance of the gold standard test.

3) Related to this, the yield of the conventional gold standard tests, which are AFB smear and TB culture, is still very low. If these were the only tests considered as part of the composite gold standard, then it would have been useless to proceed with the logistic regression. Some techniques that may be used to improve the yield of AFB staining include staining the clot that forms in standing CSF and spinning down the CSF sediment onto a slide for microscopic examination. In 1979, Kennedy and Fallon [10] showed that staining multiple samples of CSF enhanced the sensitivity to 86%.

4) Finally, the presence of HIV co-infection was not tested. As mentioned earlier, a high rate of co-infection with HIV may affect the clinical presentation and affect the choice of clinical and laboratory features to be studied.

## Conclusions

Thus we conclude that only abnormal CSF (lymphocytic pleocytosis + decreased glucose + increased protein) is associated with a diagnosis of TBM in patients with a chronic meningitis syndrome.

We recommend that these results be validated in a larger sample of patients with chronic meningitis; also that the current gold standard be improved. The yield of AFB smear and culture should be improved by proper training of medical technologists to do more meticulous examination of a larger sample, while new and more sensitive methods for TB culture can be developed.

At the same time more definite and objective criteria for basal meningeal enhancement on contrast CT scan of the head should be developed, while consistency and inter- and intra-rater variability among readers should be checked.

Lastly HIV co-infection should be checked, now that the prevalence of HIV in the country is reported to be increasing and may perhaps affect the clinical presentation and prevalence of TBM. While a definitive diagnostic laboratory exam is not yet a reality, we believe that a consensus definition of TBM should be formulated by clinicians and experts, and this will ultimately be most useful in coming up with a definition of TBM that will be relevant for subsequent researchers and clinicians. The finding of abnormal CSF can greatly aid clinicians and researchers who will opt to use a consensus panel method for determining whether a particular patient has TBM, and greatly aid subsequent management.

## Competing interests

The author declares that they have no competing interests.

## Authors' contributions

The author conceived the design of the study, carried out the data analysis, and wrote the manuscript.
